# Transient receptor potential melastatin 4 channel contributes to migration of androgen-insensitive prostate cancer cells

**DOI:** 10.18632/oncotarget.6157

**Published:** 2015-10-19

**Authors:** Christian Holzmann, Sven Kappel, Tatiana Kilch, Marcus Martin Jochum, Sabine Katharina Urban, Volker Jung, Michael Stöckle, Karen Rother, Markus Greiner, Christine Peinelt

**Affiliations:** ^1^ Biophysics, Center for Integrative Physiology and Molecular Medicine, School of Medicine, Saarland University, Homburg, Germany; ^2^ Center of Human and Molecular Biology, Saarland University, Homburg, Germany; ^3^ Clinics of Urology and Pediatric Urology, Saarland University, Homburg, Germany; ^4^ Department of Medical Biochemistry and Molecular Biology, Saarland University, Homburg, Germany

**Keywords:** prostate cancer, cancer driver gene, transient receptor potential melastatin 4 channel (TRPM4), cell migration, Ca^2+^ signaling

## Abstract

Impaired Ca^2+^ signaling in prostate cancer contributes to several cancer hallmarks, such as enhanced proliferation and migration and a decreased ability to induce apoptosis. Na^+^ influx via transient receptor potential melastatin 4 channel (TRPM4) can reduce store-operated Ca^2+^ entry (SOCE) by decreasing the driving force for Ca^2+^. In patients with prostate cancer, gene expression of TRPM4 is elevated. Recently, *TRPM4* was identified as a cancer driver gene in androgen-insensitive prostate cancer.

We investigated TRPM4 protein expression in cancer tissue samples from 20 patients with prostate cancer. We found elevated TRPM4 protein levels in prostatic intraepithelial neoplasia (PIN) and prostate cancer tissue compared to healthy tissue. In primary human prostate epithelial cells (hPEC) from healthy tissue and in the androgen-insensitive prostate cancer cell lines DU145 and PC3, TRPM4 mediated large Na^+^ currents. We demonstrated significantly increased SOCE after siRNA targeting of TRPM4 in hPEC and DU145 cells. In addition, knockdown of TRPM4 reduced migration but not proliferation of DU145 and PC3 cells. Taken together, our data identify TRPM4 as a regulator of SOCE in hPEC and DU145 cells, demonstrate a role for TRPM4 in cancer cell migration and suggest that TRPM4 is a promising potential therapeutic target.

## INTRODUCTION

In prostate cancer, decreased store-operated Ca^2+^ entry (SOCE) signals contribute to several hallmark functions of cancer, such as increased proliferation and migration and a reduction in the ability to induce apoptosis [1-4]. In addition, diminished Ca^2+^ signals impair degradation of the androgen receptor, a well-known target in prostate cancer chemotherapy [5]. Therefore, potential therapies that target Ca^2+^ homeostasis, including the SOCE pathway, are under active investigation [6]. The molecular key players of SOCE are STIM1 (stromal interaction molecule 1) in the membrane of intracellular Ca^2+^ stores and Orai1 Ca^2+^ channels in the plasma membrane. Upon Ca^2+^ release from intracellular Ca^2+^ stores, Ca^2+^ dissociates from a luminal EF hand motif of STIM1. Consequently, STIM1 proteins cluster and recruit Orai1 Ca^2+^ channels, which subsequently mediate SOCE [7, 8]. STIM/Orai-mediated Ca^2+^ signaling contributes to cell migration in different types of cancer, including melanoma [9, 10], glioblastoma [11, 12], renal carcinoma [13], hepatocarcinoma [14], breast cancer [15, 16], cervical cancer [17], and prostate cancer [18].

Transient receptor potential melastatin 4 channel (TRPM4) is a monovalent non-selective cation channel that is activated upon elevation of intracellular Ca^2+^ [19, 20]. TRPM4 initiates an important feedback mechanism for intracellular Ca^2+^ signals, as Na^+^ influx via TRPM4 can depolarize the plasma membrane potential and thus reduce the driving force for Ca^2+^ influx [19, 21]. TRPM4 contributes functionally to the pathophysiology of several cardiac diseases [22-33], migration of immune and vascular endothelial cells [34-36], and proliferation of breast cancer cells [37]. TRPM4 is expressed throughout various tissues, and expression levels are most pronounced in the colon and prostate [19, 20]. TRPM4 was significantly elevated in cancer samples in seven of nine studies that compared TRPM4 mRNA expression levels in normal prostate gland and prostate tumor tissue (*p* ≤ 0.01; www.oncomine.org, all nine studies are summarized in [38]). Another study found increased levels of TRPM4 mRNA when human prostatic intraepithelial neoplasia (PIN; abnormal but somewhat premalignant cells) develop into prostate cancer cells [39]. A recent publication by Schinke and colleagues demonstrated elevated levels of TRPM4 in androgen-insensitive prostate cancer cells and suggested a role for *TRPM4* as a cancer driver gene [38].

Thus far, the role of TRPM4 in prostate cancer has been unclear. In the present study, we investigated TRPM4 protein levels in human prostate tissue scored with the Gleason grading system. We also functionally characterized TRPM4 in primary human prostate epithelial cells (hPEC) and in androgen-sensitive (LNCaP) and androgen-insensitive (DU145 and PC3) prostate cancer cell lines. Finally, we determined the potential of TRPM4 to limit SOCE and the functional role of TRPM4 in cell migration and proliferation of prostate cancer cells.

## RESULTS

### Elevated TRPM4 expression in PIN and prostate cancer cells

**Figure 1:**
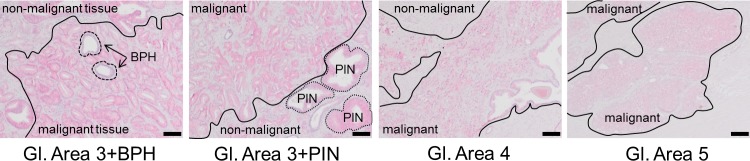
Immunohistochemical detection of TRPM4 in prostate cancer tissue Representative images (original magnification, 100×) of TRPM4-immunostained tissue sections from prostate carcinomas are shown. Red staining indicates TRPM4-positive areas. Scale bars represent 100 μm.

Prostate tissue samples contain different cell types, including fibroblasts and basal, luminal, and secretory epithelial cells, as well as neuroendocrine cells. Due to this heterogeneous nature, a comparison of the mRNA levels of a gene in cancer versus normal tissue samples is not entirely useful. We thus evaluated TRPM4 antibody staining in paraffin-embedded human prostate cancer tissues from 20 patients (Figure 1, Supplementary Table 1). Figure 1 shows examples of immunohistochemical stainings with TRPM4-specific antibody of prostate cancer tissue samples that were rated with different Gleason scores. We found expression of TRPM4 in hPEC and weak or negligible expression in stromal cells (Figure 1). Areas identified as non-malignant (e.g. Figure 1 panel 1, upper left and panel 2, bottom right) or benign prostatic hyperplasia (BPH) showed no or faint TRPM4 immunoreactivity. In areas of PIN or increased Gleason growth patterns, medium or strong signal intensity of TRPM4 was detected (Figure 1). The specificity of the TRPM4 antibody was verified in consecutive tissue slices (α-TRPM4 and control) and with Western blot analysis upon an siRNA based knockdown of TRPM4 in LNCaP and DU145 cells (SFigure 1A and 1B). Supplementary Table 1 summarizes the clinical characteristics (i.e., classification by Gleason score and tumor nodes metastasis [TNM] stage) of 20 patients with prostate cancer. For these patients, TRPM4 signal intensities were evaluated in areas of tumor, PIN, and BPH. We did not observe any correlation between TRPM4 staining and the clinical or pathological stages of prostate cancer. In all tissue samples, we detected strong TRPM4 staining in malignant and PIN areas and weak or absent staining in areas with BPH.

### In hPEC, TRPM4 generates large Na^+^ currents and limits SOCE

**Figure 2:**
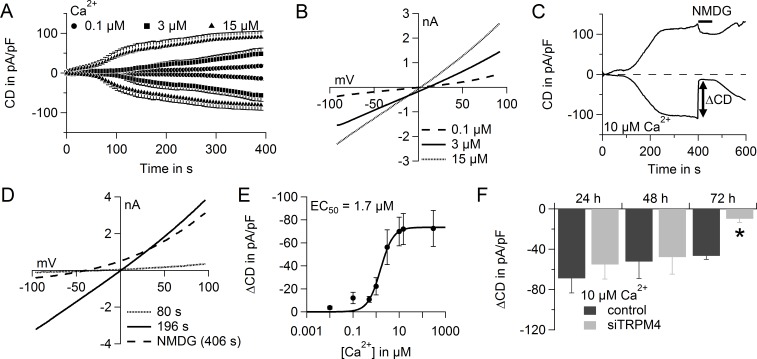
TRPM4 currents in hPEC **A.** Time course of averaged TRPM4 current densities (CD) evoked by adjusted intracellular Ca^2+^ concentrations as indicated (100 nM Ca^2+^, *n* = 7; 3 μM Ca^2+^, *n* = 12; and 15 μM Ca^2+^, *n* = 11). **B.** Average IV for cells in **A.** at time = 396 s. **C.** TRPM4 current over time in a typical cell when NMDG was applied as indicated. **D.** IV for the typical cell in **C.** at indicated time points. **E.** Dose-response curve for TRPM4 currents induced with various Ca^2+^ concentrations: 10 nM Ca^2+^, *n* = 8; 500 nM Ca^2+^, *n* = 6; 1 μM Ca^2+^, *n* = 8; 10 μM Ca^2+^, *n* = 10; and 300 μM Ca^2+^, *n* = 7. Same cells as in **A.** for 100 nM, 3 μM, and 15 μM Ca^2+^. EC_50_ for Ca^2+^ was 1.7 μM. **F.** ΔCurrent density (ΔCD) of TRPM4 current 24 h, 48 h, and 72 h after siRNA-based knockdown of TRPM4 (light grey bars: 24 h, *n* = 7; 48 h, *n* = 9; and 72 h, *n* = 11) and control RNA treatment (dark grey bars: 24 h, *n* = 5; 48 h, *n* = 5; and 72 h, *n* = 9).

TRPM4 plays a role in several physiological functions such as membrane depolarization, cell death, migration and proliferation [26, 40]. To functionally determine Na^+^ currents via TRPM4 in hPEC, we performed whole-cell patch clamp analysis and evoked TRPM4 currents with 0.1-15 μM free Ca^2+^ in the patch pipette. Intracellular Ca^2+^ activated TRPM4 currents in hPEC in a dose-dependent manner (Figure 2A; current-voltage curves [IV] are shown in Figure 2B). For further analysis, we corrected for current not specific to TRPM4, which can also be activated by intracellular Ca^2+^ e.g. Ca^2+^ activated Cl^−^ channels [41]. To do so, after current development (400 s), Na^+^ in the external bath solution was replaced by NMDG, a nonpermeable cation. This abolishes all Na^+^ inward current, and the net Na^+^ current density (ΔCD) can be analyzed by subtracting NMDG currents (at 406 s) from currents in Na^+^ (at 396 s). An example of calculating ΔCD during analysis of a typical cell is shown in Figure 2C, and the corresponding IV are shown in Figure 2D. 10 μM Ca^2+^ evoked maximal ΔCD and we calculated an EC_50_ for Ca^2+^-dependent TRPM4 activation of 1.7 ± 0.2 μM, which is consistent with published EC_50_ values of 0.4-9.8 μM [19, 42-44] (Figure 2E). In an siRNA-based knockdown, we found a significant reduction in TRPM4 currents 72 h after transfection (Figure 2F). Relative gene expression levels of TRPM4 normalized to TATA box binding protein (TBP) were reduced 72 h after knockdown, as shown by RT-PCR (Supplementary Figure 1C). Taken together, these experiments demonstrate that increasing intracellular Ca^2+^ concentrations generate large TRPM4-mediated Na^+^ currents (up to approximately 70 pA/pF) in hPEC.

**Figure 3:**
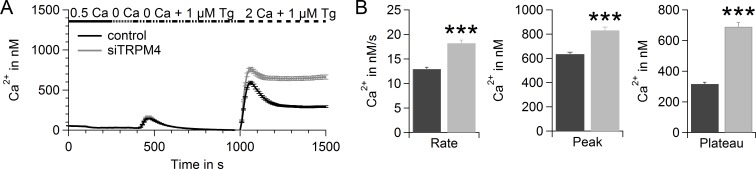
TRPM4 limits SOCE in hPEC **A.** Thapsigargin-induced SOCE in hPEC transfected with control RNA (black, *n* = 338; two donors) or TRPM4 siRNA (grey, *n* = 265; two donors) 72 h after transfection measured in a Fura-2-based Ca^2+^ readdition protocol. **B.** Analysis of the rate, peak, and plateau of Ca^2+^ entry of cells shown in **A**.

In a fluorescence-based Fura-2 Ca^2+^ assay, we tested the potential of TRPM4 to reduce SOCE in hPEC (Figure 3A), as a large Na^+^ influx can lower the driving force for Ca^2+^. Upon addition of thapsigargin (Tg), an inhibitor of sarcoplasmic/endoplasmic reticulum Ca^2+^ ATPase (SERCA), intracellular Ca^2+^ stores were depleted. The Ca^2+^ release from intracellular Ca^2+^ stores was detected as a small increase in intracellular Ca^2+^ (400-600 s). Upon readdition of extracellular Ca^2+^ (time > 1000 s), SOCE was recorded as described previously [45]. After TRPM4 knockdown, we detected elevated SOCE in hPEC (Figure 3A). The significant increase in rate, peak, and plateau of SOCE after knockdown demonstrates a clear role for TRPM4 in limiting SOCE signals in hPEC (Figure 3B).

### TRPM4 generates large Na^+^ currents in prostate cancer cells and limits SOCE in DU145

**Figure 4:**
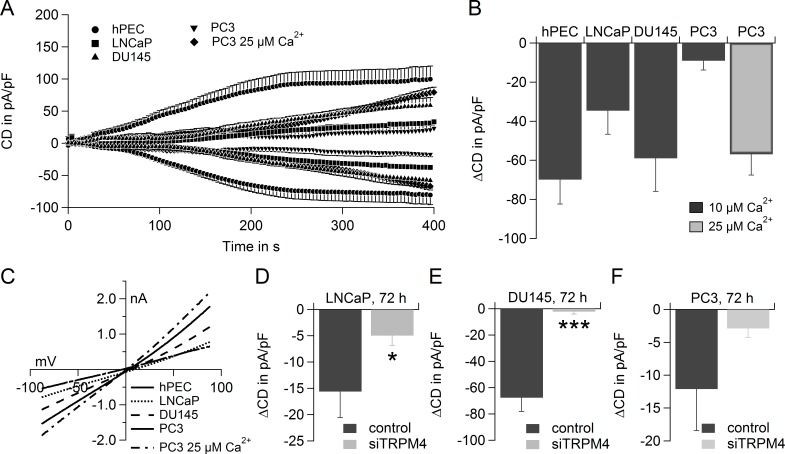
TRPM4 currents in hPEC, LNCaP, DU145 and PC3 **A.** Time course of averaged TRPM4 current densities (CD) in hPEC, LNCaP, DU145 and PC3 evoked by 10 μM Ca^2+^ if not indicated otherwise, hPEC (*n* = 10, same cells as in **2E**., LNCaP (*n* = 10), DU145 (*n* =7) and PC3 (*n* = 6 for 10 μM Ca^2+^ and *n* = 5 for 25 μM Ca^2+^). **B.** ΔCD from cells in **A.**. **C.** IV at t = 396 s for cells in **A.**. **D.** ΔCD evoked with 10 μM Ca^2+^ in the patch pipette in LNCaP cells transfected with control RNA (dark grey bar, *n* = 5) or siRNA to down-regulate TRPM4 (light grey bar, *n* = 6). **E.** ΔCD evoked with 10 μM Ca^2+^ in the patch pipette in DU145 cells transfected with control RNA (dark grey bar, *n* = 5) or siRNA to down-regulate TRPM4 (light grey bar, *n* = 7). **F.** ΔCD evoked with 10 μM Ca^2+^ in the patch pipette in PC3 cells transfected with control RNA (dark grey bar, *n* = 5) or siRNA to down-regulate TRPM4 (light grey bar, *n* = 5).

We compared TRPM4 activity in hPEC, as well as in the prostate cancer cell lines LNCaP, DU145 and PC3. TRPM4 currents were activated with 10 μM intracellular Ca^2+^, and in case of the PC3 cells 25 μM Ca^2+^ were used in addition (Figure 4A and 4B, IV are shown in Figure 4C). TRPM4-nonspecific currents were subtracted as described above. Whereas in hPEC TRPM4 exhibited currents of 70 ± 13 pA/pF, in the prostate cancer cell lines, the TRPM4 current size was 35 ± 12 pA/pF in LNCaP, 59 ± 17 pA/pF in DU145, 9 ± 5 pA/pF in PC3 cells for 10 μM Ca^2+^ and 56 ± 11 pA/pF in PC3 cells for 25 μM Ca^2+^ (Figure 4B). The corresponding IV are shown in Supplementary Figure 2A-2C. While in hPEC 10 μM intracellular Ca^2+^ evoked maximal current (Figure 2E), current development in cancer cells is slower and can be further enlarged with increasing intracellular Ca^2+^ concentrations as has been demonstrated here for PC3 cells (Figure 4A and 4B).

siRNA-based knockdown of TRPM4 significantly reduced the current size in LNCaP (Figure 4D; IV are shown in Supplementary Figure 3A) and DU145 (Figure 4E; IV are shown in Supplementary Figure 3B), and reduced current size in PC3 (Figure 4F; IV are shown in Supplementary Figure 3C) demonstrating the TRPM4-specificity of the detected currents in prostate cancer cells. Knockdown efficiency after 72 h was determined by RT-PCR, as shown in Supplementary Figure 4A-4C. We also controlled for off-target effects of the TRPM4 specific siRNA within the SOCE pathway and found that expression levels of Orai1, Orai2, Orai3, STIM1 and STIM2 remain largely unchanged (Supplementary Figure 4D and 4E).

As in human prostate cancer tissue TRPM4 was up-regulated (Figure 1); in native cancer cells, TRPM4 currents might well exceed TRPM4 currents in hPEC, even though under the conditions used here TRPM4 currents in cancer cell lines were lower than in hPEC.

**Figure 5:**
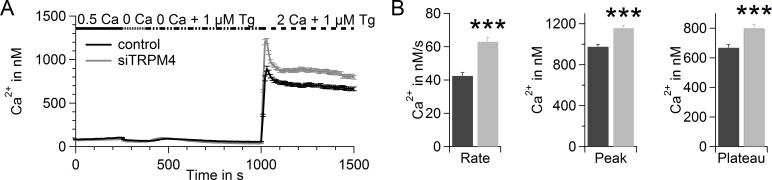
TRPM4 limits SOCE in in DU145 **A.** Thapsigargin-induced SOCE in DU145 transfected with control RNA (black, *n* = 338) or TRPM4 siRNA (grey, *n* = 215) measured during a Ca^2+^-readdition protocol 72 h after transfection. **B.** Analysis of the rate, peak, and plateau of Ca^2+^ entry of cells shown in **A.**.

To further delineate the role of TRPM4 in prostate cancer, we investigated the potential of TRPM4 to limit Ca^2+^ signals in the prostate cancer cell lines DU145 and PC3. We performed the same Fura-2 based Ca^2+^ assay as in Figure 3A for DU145 (Figure 5A). In DU145 cells, knockdown of TRPM4 significantly increased the rate, peak, and plateau of SOCE (Figure 5A and 5B). When the membrane potential is clamped close to 0 mV (high K^+^ Ringer), knockdown of TRPM4 only slightly increase the peak of SOCE while the Ca^2+^ entry rate and plateau of SOCE remained unchanged (SFigure 5A). Under these conditions the relative increase in SOCE upon knockdown of TRPM4 is significantly reduced for the Ca^2+^ entry rate and plateau of SOCE (SFigure 5B) compared to standard conditions. We conclude that upon activation of TRPM4 the concomitant Na^+^ influx leads to a depolarization of the membrane potential. Thus the driving force for Ca^2+^ entry is reduced and SOCE is limited. In PC3 cells we did not detect increased SOCE upon knockdown of TRPM4 (SFigure 6).

Both, DU145 and PC3 cells exhibit large TRPM4 currents and in addition TRPM4 significantly reduced SOCE in DU145 cells. Thus, we investigate the function of TRPM4 in androgen-insensitive prostate cancer cells DU145 and PC3.

### TRPM4 in migration and proliferation

**Figure 6:**
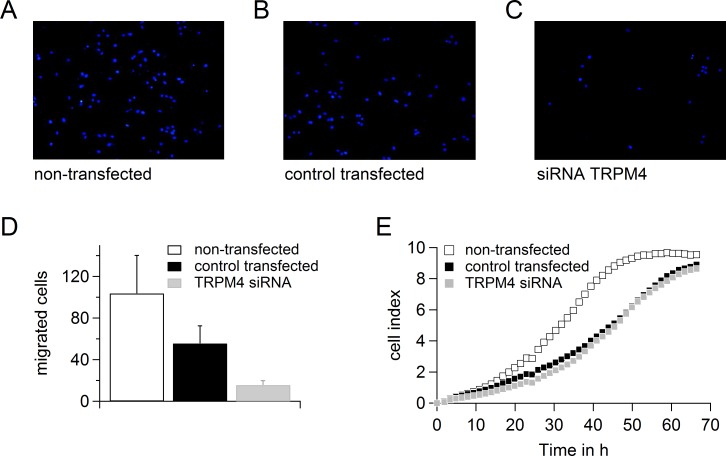
Down-regulation of TRPM4 leads to reduced migration potential of DU145 cells **A.**-**C.** Migrated cells when DU145 cells were either not transfected **A.** or transfected with control RNA **B.** or siRNA against TRPM4 **C.**. **D.** Analysis of migration of cells in (**A**.-**C**.). **E.** Proliferation of cells in (**A**.-**C**.).

**Figure 7:**
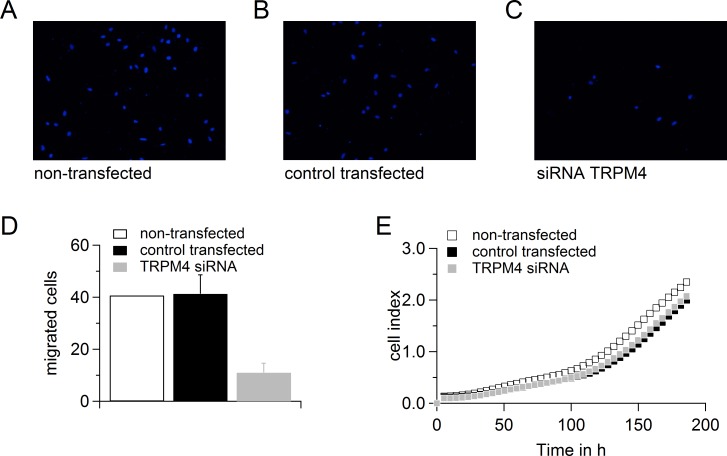
Down-regulation of TRPM4 leads to reduced migration potential of PC3 cells **A.**-**C.** Migrated cells when PC3 cells were either not transfected **A.** or transfected with control RNA **B.** or siRNA against TRPM4**C.**. **D.** Analysis of migration of cells in (**A**.-**C**.). **E.** Proliferation of cells in (**A**.-**C**.).

We tested the migration of DU145 and PC3 cells when cells were either non-transfected or transfected with control RNA or siRNA targeting TRPM4 (Figure 6A-6D and 7A-7D). Although transfection had a clear effect on the migration potential of these cells, knockdown of TRPM4 resulted in a strong decrease in migration compared to control transfected cells (Figure 6D and Figure 7D). Figure 6E and 7E show that transfection affected the proliferation of the DU145 and PC3 cells, but there was no difference between TRPM4 siRNA- or control RNA-transfected cells. To summarize, the decreased migration of transfected DU145 and PC3 cells upon control transfection can be explained by decreased proliferation, but knockdown of TRPM4 also specifically reduces the migration of DU145 and PC3 cells.

## DISCUSSION

Cell migration depends on intracellular Ca^2+^ and lately the spatial and temporal organisation of STIM/Orai dependent Ca^2+^ gradients has been revealed [46-48]. In addition to SOCE channels, several TRP channels contribute to the migration of cancer cells, including TRP channels TRPC1, TRPM7, TRPM8, TRPV1, TRPV2, and TRPV6 [3]. TRPM4 is known to generate an important feedback mechanism for SOCE and to contribute to the migration of dendritic cells, mast cells and vascular endothelial cells [19, 21, 34-36, 49].

We demonstrated that TRPM4 protein levels in areas of PIN and human prostate cancer tissues rated with different Gleason scores were elevated compared to TRPM4 expression in healthy tissue or areas of BPH. These findings are in line with several studies that demonstrate increased levels of TRPM4 mRNA in prostate cancer samples compared to healthy tissues (summarized in [38]).

TRPM4 conducted large Na^+^ currents in both, hPEC and in prostate cancer cell lines. We here investigated the role of TRPM4 in prostate cancer cells that - in contrast to hPEC - are characterized by several pathophysiological changes in cellular functions such as unlimited proliferation and enhanced migration potential. Even though the current in DU145 and PC3 cells may be lower than in native cancer cells, here, DU145 and PC3 serve as model cell lines to investigate the function of TRPM4 in androgen-insensitive prostate cancer. In both prostate cancer cell lines knockdown of TPRM4 reduced migration while proliferation remained unaffected.

In DU145 cells activation of TRPM4 depolarizes the membrane potential, reduces the driving force for Ca^2+^ and is thus an important negative feedback regulator of SOCE. While TRPM4 regulates SOCE in DU145 cells we could not detect a regulatory role for TRPM4 on Ca^2+^ signaling in PC3 cells, probably due to the slower current activation and/or limitations of our Ca^2+^ imaging protocol. A very recent study describes TRPM4 as a component of the adhesome required for migration. TRPM4 contributes to cell migration not only by regulating intracellular Ca^2+^ signaling but also by direct interaction with several proteins involved in actin cytoskeleton dynamics [50]. Thus the regulatory mechanism of TRPM4 on migration of prostate cancer cells may differ in DU145 and PC3 cells and include Ca^2+^ independent mechanisms.

While Armisen et al. clearly demonstrate a role for TRPM4 in the proliferation of breast cancer cells [37], we cannot detect a TRPM4-specific effect on proliferation of prostate cancer cells. One explanation may be that the transfection of prostate cancer cells itself has a clear impact on their proliferation which may cover any specific contribution of TRPM4. Another explanation could be that TRPM4 regulates proliferation in breast cancer cells while in prostate cancer TRPM4 does not impair proliferation.

Disordered Ca^2+^ signals in cancer have been investigated for decades, and consequently several therapeutic strategies have been developed to target Ca^2+^-transporting enzymes [4, 6]. Currently, the prostate-targeted thapsigargin derivative mipsagargin is being tested in a phase II clinical trial [51]. Advanced strategies based on calmodulin antagonists that further sensitize prostate cancer cells to thapsigargin therapies have delivered promising results [52].

Down-regulation of TRPM4 strongly decreased cell migration of DU145 and PC3 cells, suggesting a role for TRPM4 in prostate cancer invasion—the initial step for tumor metastasis. We suggest that up-regulation of the TRPM4 cancer driver gene [38] functionally contributes to the development of PIN and prostate cancer by elevating the migration potential of androgen-insensitive prostate cancer cells. TRPM4 contributes to the migration of prostate cancer cells and is thus an interesting potential pharmacological target. The ubiquitous expression pattern of TRPM4 implies a versatile role for TRPM4 in cancer cell migration.

## MATERIALS AND METHODS

### Immunohistochemistry

This study was approved by the local ethical review board and was performed in accordance with the Declaration of Helsinki. After we received written informed consent, prostate cancer tissues from 20 patients were obtained after radical prostatectomy from thus far untreated prostate cancer patients. Gleason score, pathologic stage, histologic diagnosis, and tumor node metastasis classification (TNM) were done in accordance with the guidelines of the Union for International Cancer Control (2002). Tissue samples were cut into 3 μm thick sections, transferred onto Superfrost Ultra Plus Microscope Slides (Menzel-Gläser, Braunschweig, Germany), and dried in an incubator at 37°C overnight. After deparaffinization, heat-induced epitope retrieval was performed by treatment in 10 mmol/L citrate buffer (pH 6.0) and nonspecific protein binding sites were blocked by incubation in blocking solution (80 mL of 0.1M Tris-HCl pH 7.2, 3 g of bovine serum albumin [BSA; Sigma Aldrich, Germany], and 20 mL of fetal bovine serum [FBS; Sigma Aldrich Chemie GmbH]) for 30 min at room temperature. Subsequently, primary antibody incubation was performed with a 1:100 solution (diluted in phosphate-buffered saline/0.3% BSA) of a specific TRPM4 monoclonal mouse antibody ([53] Clone 10H5, TA500381; Origene, Rockville, MD, USA) overnight at 4°C. Each staining series included negative controls without α-TRPM4 incubation. Visualization was performed with the DAKO Real Detection System (DAKO, Glostrup, Denmark) in accordance with the manufacturer's instructions, and slides were counterstained with hematoxylin. TRPM4 staining intensity was classified as no staining (0), weak (+), intermediate (++), or strong (+++).

### Cell culture and prostate tissue collection

Lymph node carcinoma of the prostate (LNCaP) cells, DU145 and PC3 were purchased from the American Type Cell Culture Collection (ATCC, Rockville, MD, USA) and cultured in RPMI Medium 1640 (Life Technologies) supplemented with 10% FCS and 1% penicillin/streptomycin (Life Technologies). Prostate tissue was obtained from prostectomy specimens (ethics approval 168/05, Ärztekammer des Saarlandes), and hPEC were isolated, with slight modifications, in accordance with [54], as described in [45].

### Western blot analysis

For Western blot analysis, cells were lysed and 200 μg of total protein was separated by 10% SDS-PAGE. Immunoblots were probed with anti-TRPM4 antibody (1:250; Origene). The manusfacturer's control was used to test for positive TRPM4 staining. Antibodies and proteins were detected with an enhanced chemiluminescence detection system (ECL; Biorad).

### Quantitative real-time PCR (qRT-PCR)

Total RNA from LNCaP, DU145, PC3 and hPEC was isolated with TRIzol Reagent (Life Technologies) and from prostate tissue with the RNeasy Mini kit (Qiagen). For reverse transcription, 0.8 μg of isolated total RNA was used. Complementary DNA (cDNA; 0.5 μl) and 300 nM primer were used with a QuantiTect SYBRgreen kit (Qiagen). PCR conditions were as follows: 15 min at 95°C; 45 cycles of 30 s at 95°C, 45 s at 58°C, and 30 s at 72°C; and finally a cycle (60 s at 95°C, 30 s at 55°C, and 30 s at 95°C) to determine specificity by a dissociation curve using the MX3000 cycler (Stratagene). Expression of target genes was normalized to expression of the reference genes RNA polymerase II (RNAPol, NM_000937) and/or TATA box binding protein (TBP, NM_003194). Primer sequences for TRPM4 (NM_017636) were 5′-gtatctgctctcggacaag-3′ (forward) and 5′-aagagctgaggaaactgcat-3′ (reverse).

### Ca^2+^ imaging

Ca^2+^ imaging experiments were performed in accordance with [55]. Cells were cultured overnight on glass cover slips and loaded with 1 μM Fura-2/AM at 37°C for 20 min. Glass coverslips were placed in a perfusion chamber in a Zeiss Axio Observer.A1 fluorescence microscope equipped with a “Plan-Neofluar” 20×/0.4 objective (Zeiss). The excitation light, generated by a Polychrome V in a TILL Photonics real-time imaging system, alternated at 340 and 380 nm, and the exposure time was set to 50 ms in each channel. Light intensity at emission wavelength 440 nm was detected every 5 s and digitized by a charge-coupled device camera (Q-Imaging Retiga 2000RV). Bath solution contained (in mM): 155 NaCl, 4.5 KCl, 2 MgCl_2_, 10 D-glucose, and 5 Hepes (pH 7.4 with NaOH). CaCl_2_ and thapsigargin in the bath solution was adjusted as indicated. Stock solutions of thapsigargin were prepared in DMSO at a concentration of 1 mM. In the experiment in SFigure 5 the bath solution contained (in mM): 155 KCl, 2 MgCl_2_, 10 D-glucose, and 5 Hepes (pH 7.4 with NaOH).

### Electrophysiology

Cells were patched in a tight-seal whole-cell configuration as described in [56, 57]. Every 2 s, voltage ramps of 50 ms duration from a holding potential of 0 mV, spanning −100 mV to +100 mV, were delivered by a Patchmaster software-controlled EPC-10 patch-clamp amplifier (HEKA). Before each voltage ramp, capacitive currents were determined and corrected. Data were filtered at 1 kHz and sample rate was 3 kHz, and the liquid junction potential was corrected (−10 mV). For analysis, currents were extracted at −80 mV and 80 mV, normalized to the cell capacity, and plotted versus time. Bath solutions contained (in mM): 140 NaCl, 0.5 CaCl_2_, 3 MgCl_2_, 10 HEPES, and 30 glucose. pH was adjusted with NaOH to 7.2, and osmolarity was 330 mOsmol/L. In the N-methyl-d-glucamine (NMDG) bath solution, 140 mM NaCl was replaced by 140 mM NMDG. Pipette solution contained (in mM): 140 Cs glutamate, 10 HEDTA, 10 HEPES, 8 NaCl, MgCl_2,_ and CaCl_2_. MgCl_2_ and CaCl_2_ were calculated (http://www.stanford.edu/­wcpatton/­webmaxcS.htm) and added to reach an end concentration for free Mg^2+^ of 3 mM and free Ca^2+^ as indicated.

### Data analysis and statistics

Data are given as means ± standard error of the mean (SEM) and were analyzed with TILLVision (TILL Photonics), Fitmaster 2.35 (HEKA), Igor Pro (Wavemetrics), and Microsoft Excel (Microsoft). Asterisks indicate significance and were determined by unpaired, two-sided Student's *t*-test: *, *p* < 0.05; **, *p* < 0.01; and ***, *p* < 0.001. Half maximal effective concentration (EC_50_) was determined by a fit with Hill's equations (least-squares method). For Ca^2+^ imaging, intracellular Ca^2+^ concentration was calculated from the equation [Ca^2+^]_i_ = K(R−R_min_)/(R_max_-R), in which K, R, R_min_, and R_max_ were determined in the corresponding in situ calibration for hPEC, LNCaP, and DU145 cells, in accordance with [58]. For qRT-PCR, relative expression was calculated with the ΔCq method (2^−ΔCq^) and Cq values were determined with the MX3000.

### Small interfering RNA transfection (siRNA)

TRPM4 siRNAs were 5′-GCACGACGUUC AUAGUUGATT-3′ (sense) and 3-′GACGUGCUGCAA GUAUCAACU-5′ (antisense) (Qiagen). Non-silencing RNA were MS_control_mod [sense: 5′OmeA-OMeA-AGGUAGUGUAAUCGCd(CUU) OMeG-OmeT-OMeT3′; antisense: 3′;OmeT-OmeTUCCAUCACA UUAGCGGAAdC5′]. Nucleofector II (Amaxa Lonza) nucleofector and the Nucleofector Transfection Kit R (Lonza) were used in accordance with the manufacturer's instructions to transfect 0.12 nmol siRNA per transfection.

### Migration potential analysis

Migration was tested with the BD Falcon FluoroBloksystem (BD Biosciences, Franklin Lakes, NJ, USA) in 24-well inserts. A total of 2.5 × 10^4^ DU 145 or PC3 cells treated with control RNA or TRPM4 siRNA were loaded into this system in RPMI-1640 medium containing 1.0% FBS. The inserts were placed in RPMI-1640 medium with 10% FBS as an attractant. After 48 h, the cells were fixed with methanol and stained with DAPI and migrating cells were analyzed on the backside of the membrane by fluorescence. The experiment was repeated three times, and the migrated cells of at least three individual images were automatically counted using NIS-Elements AR Software (Nikon).

### Real-time cell proliferation analysis

The xCELLigence SP system (Roche Diagnostics GmbH, Mannheim, Germany) was used for real-time analysis of cell proliferation. In this system, 5.0 × 10^3^ DU145 cells or PC3 cells, either untransfected or transfected with control RNA or TRPM4 siRNA, were seeded into a 96-well E-plate (Roche Diagnostics GmbH) in accordance with the manufacturer's instructions. Cells pretreated with siRNA were seeded 72 h after transfection. Cell proliferation was monitored for 96 h, and data were evaluated with RTCA 2.0 software (Roche Diagnostics GmbH).

## SUPPLEMENTARY MATERIAL FIGURES AND TABLE


