# Supplementary material for: Transient receptor potential melastatin 4 channel contributes to migration of androgen-insensitive prostate cancer cells
Source: Oncotarget. 2015 Oct 19;6(39):41783–93. doi: 10.18632/oncotarget.6157 (PMC4747188; doi:10.18632/oncotarget.6157)
Supplement: Supplementary file 1 [file oncotarget-06-41783-s001.pdf]

Transient receptor potential melastatin 4 channel contributes to migration of androgen-insensitive prostate cancer cells

Supplementary Material

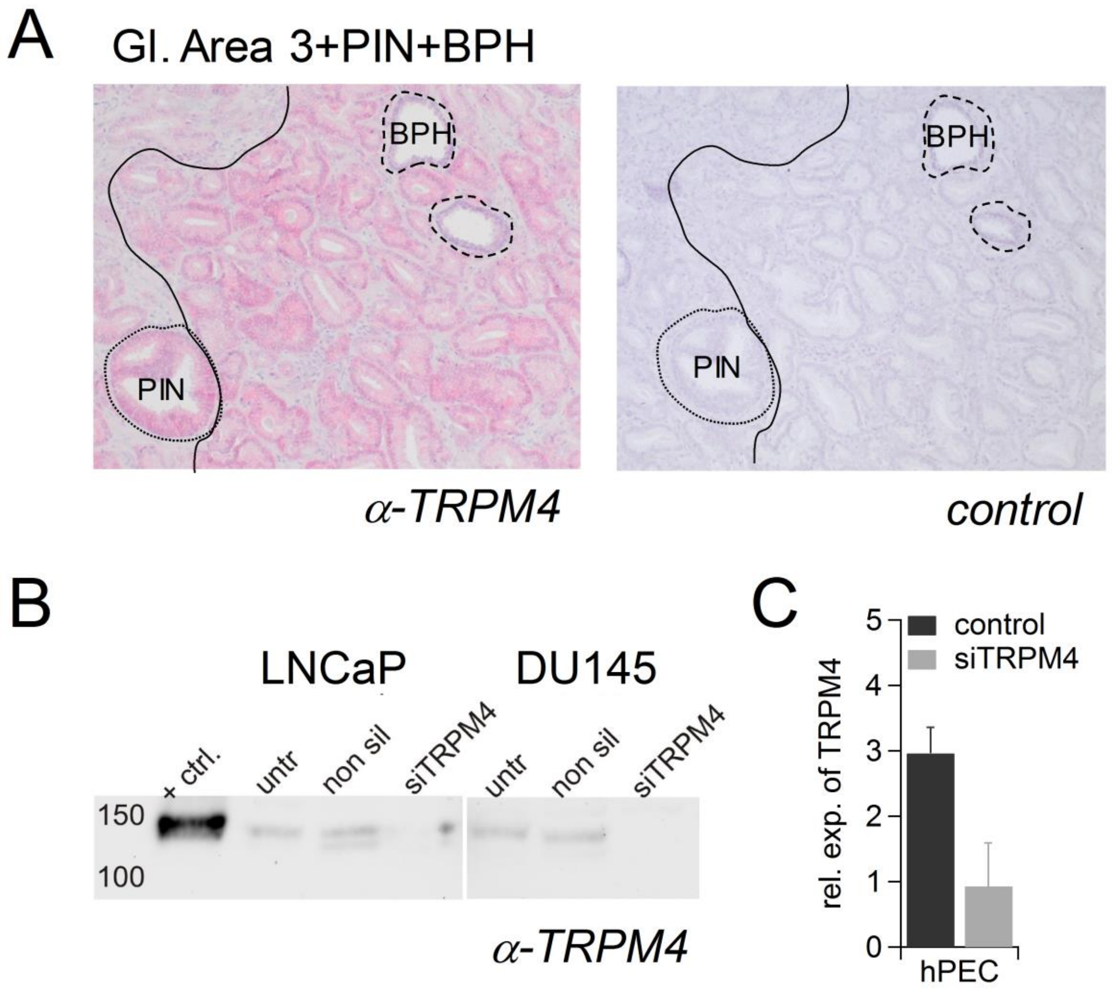

Supplementary Fig. 1: Specificity of α-TRPM4 antibody and knockdown efficiency of TRPM4 siRNA. (A) Immunofluorescence staining for TRPM4 in prostate cancer tissue or control in two consecutive tissue slices. **(B)** Western blot analysis of TRPM4 protein in LNCaP or DU145 that were non-transfected or transfected with control RNA or siRNA targeting TRPM4. **(C)** Efficiency of TRPM4 down-regulation by siRNA 72 h after transfection detected by qRT-PCR and normalized to TBP in hPEC (n = 3).

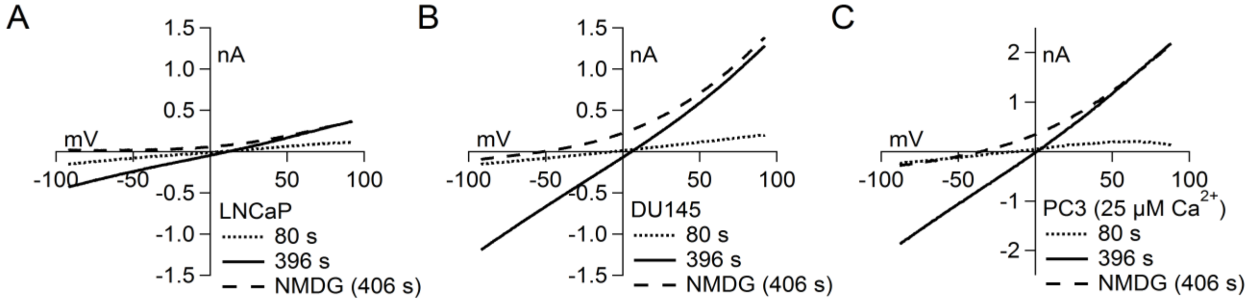

Supplementary Fig. 2: IV of TRPM4 in non-transfected cells. (A-C) IV from measurements in LNCaP, DU145 and PC3 cells shown in **Fig. 4B** extracted at indicated time points.

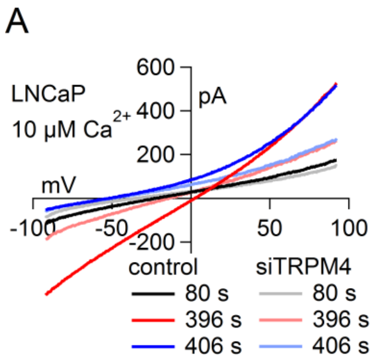

Supplementary Fig. 3: IV of TRPM4 in transfected cells. (A-C) IV from measurements in LNCaP, DU145 and PC3 cells shown in **Fig. 4D**-F extracted at indicated time points.

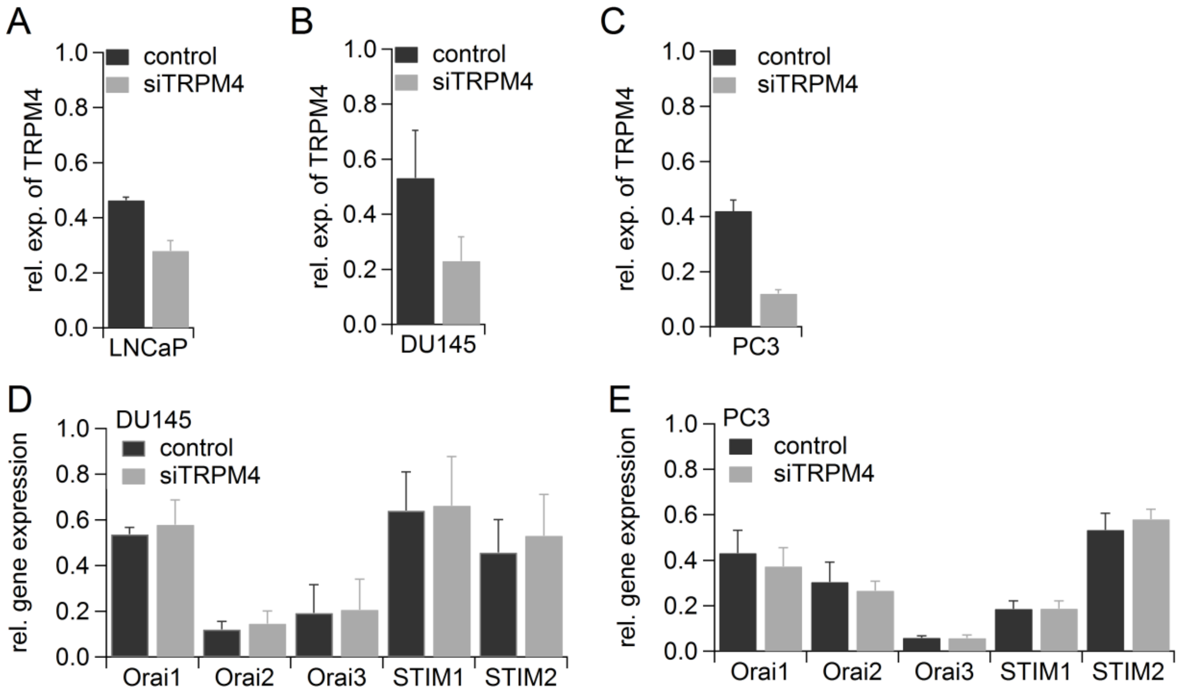

Supplementary Fig. 4: Knockdown efficiency of TRPM4 on mRNA levels and off-target effects upon knockdown. (A-C) Efficiency of TRPM4 down-regulation by siRNA 72 h after transfection when normalized to TBP in LNCaP (n = 3), DU145 (n = 3) and PC3 cells (n = 3) detected by qRT-PCR. **(D-E)** mRNA levels of Orai1, Orai2, Orai3, STIM1 and STIM2 upon knockdown of TRPM4 (n = 3).

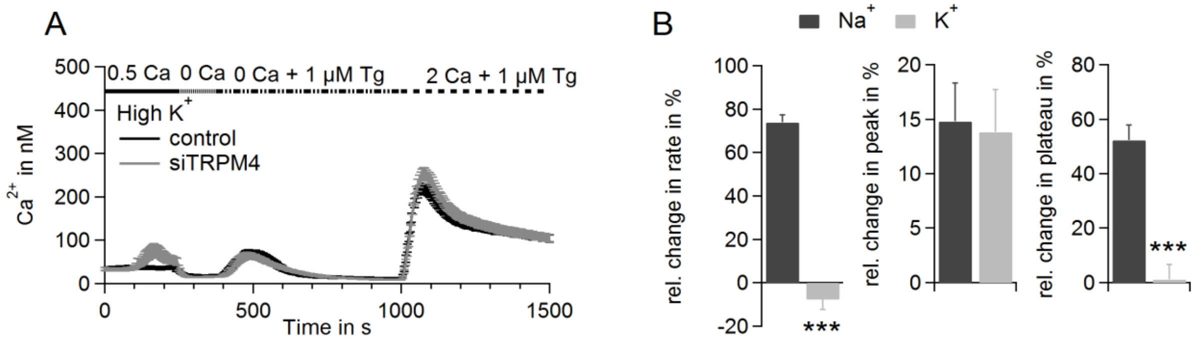

Supplementary Fig. 5: SOCE in DU145 upon knockdown of TRPM4 when external Na^+ was replaced with K^+. (A) Tg-induced SOCE in DU145 transfected with control RNA (black, *n* = 126) or TRPM4 siRNA (grey, *n* = 137) 72 h after transfection measured in a Fura-2–based Ca^{2+} readdition protocol when extracellular Na^+ was replaced by K^+. **(B)** Relative changes in Ca^{2+} entry rate, peak and plateau of SOCE upon knockdown of TPRM4 under standard conditions (Na^+, black bars, from cells in **Fig. 5A**) or when Na^+ was replaced by K^+ (grey bars, from cells in **SFig. 5A**).

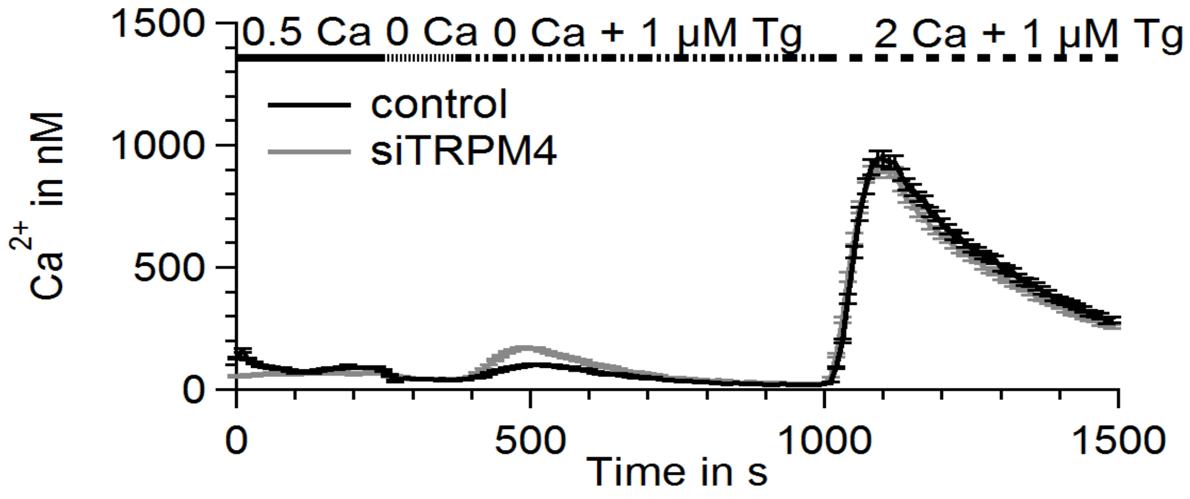

Supplementary Fig. 6: SOCE in PC3 upon knockdown of TRPM4. (A) Tg-induced SOCE in PC3 transfected with control RNA (black, *n* = 292) or TRPM4 siRNA (grey, *n* = 358) 72 h after transfection measured in a Fura-2–based Ca^{2+} readdition protocol.

Supplementary Table 1

Clinical characteristics and signal intensity of TRPM4 immune reactivity in prostate carcinoma tissue

case	Gleason Score	T stage	N	M	Tumor	PIN	BPH
PCA001	3+2=5	pT4	N0	M0	++	++	+-
PCA002	4+3=7	pT3b	N0	M0	+++	+++	n.e.
PCA003	4+3=7	pT3a	N0	M0	+++	+++	+
PCA004	4+3=7	pT3a	N0	M0	+++	+++	+
PCA005	4+3=7	pT3a	N0	M0	++	++	-
PCA006	4+3=7	pT2c	N0	M0	++	+++	-
PCA007	3+4=7	pT2c	N0	M0	+++	+++	++
PCA008	4+5=9	pT3b	N0	M0	++	+++	+
PCA009	4+3=7	pT3a(est)	N0	M0	++	++	-
PCA010	4+3=7	pT3a(est)	N0	M0	++	++	-
PCA011	3+4=7	pT2c	N0	M0	++	++	n.e.
PCA012	3+4=7	pT3a(est)	N0	M0	+++	++	n.e.
PCA013	5+4=9	pT2c	N0	M0	+++	++	-
PCA014	3+4=7	pT2c	N0	M0	+++	++	-
PC0015	3+4=7	pT2c	N0	M0	+++	++	-
PCA016	3+4=7	pT3b	N0	M0	+++	++	-
PCA017	4+4=8	pT2a	N0	M0	+++	++	+
PCA018	3+4=7	pT2c	N0	M0	+++	++	-
PCA019	3+4=7	pT2c	N0	M0	+++	+	-
PCA020	3+3=6	pT2c	N0	M0	+++	+	-
